# Use of data processing for rapid detection of the prostate-specific antigen biomarker using immunomagnetic sandwich-type sensors

**DOI:** 10.3762/bjnano.10.210

**Published:** 2019-11-06

**Authors:** Camila A Proença, Tayane A Freitas, Thaísa A Baldo, Elsa M Materón, Flávio M Shimizu, Gabriella R Ferreira, Frederico L F Soares, Ronaldo C Faria, Osvaldo N Oliveira

**Affiliations:** 1 Chemistry Department, Federal University of São Carlos, CP 676, São Carlos 13565-905, São Paulo, Brazil; 2 São Carlos Institute of Physics, University of São Paulo, CP 369, São Carlos 13560-970, São Paulo, Brazil; 3 Brazilian Nanotechnology National Laboratory (LNNano), Brazilian Center for Research in Energy and Materials (CNPEM), Campinas 13083-970, São Paulo, Brazil; 4 Carlos Institute of Chemistry, University of São Paulo, São Carlos 13560-970, São Paulo, Brazil; 5 Chemistry Department, Federal University of Paraná, Curitiba, 81531-980, Paraná, Brazil

**Keywords:** cancer biomarkers, magnetite nanoparticles, microfluidic devices, nanoarchitectonics, information visualization, sandwich-type immunosensors, screen-printed electrodes

## Abstract

Diagnosis of cancer using electroanalytical methods can be achieved at low cost and in rapid assays, but this may require the combination with data treatment for determining biomarkers in real samples. In this paper, we report an immunomagnetic nanoparticle-based microfluidic sensor (INμ-SPCE) for the amperometric detection of the prostate-specific antigen (PSA) biomarker, the data of which were treated with information visualization methods. The INμ-SPCE consists of eight working electrodes, reference and counter electrodes. On the working electrodes, magnetic nanoparticles with secondary antibodies with the enzyme horseradish peroxidase were immobilized for the indirect detection of PSA in a sandwich-type procedure. Under optimal conditions, the immunosensor could operate within a wide range from 12.5 to 1111 fg·L^−1^, with a low detection limit of 0.062 fg·L^−1^. Multidimensional projections combined with feature selection allowed for the distinction of cell lysates with different levels of PSA, in agreement with results from the traditional enzyme-linked immunosorbent assay. The approaches for immunoassays and data processing are generic, and therefore the strategies described here may provide a simple platform for clinical diagnosis of cancers and other types of diseases.

## Introduction

The prostate-specific antigen (PSA) used in clinical diagnosis is present in normal prostatic secretions, but its concentration is often elevated in prostate cancer patients. In spite of its lack of specificity, PSA screening has contributed to a significant decline (45–70%) in prostate cancer mortality since the early 1990s [1]. To identify cancer biomarkers and to develop methodologies to quantify them at low cost is critical for early cancer diagnostics, while it also helps to understand cancer diseases [1]. Protein biomarkers are commonly measured using conventional immunoassays such as enzyme-linked immuno-sorbent assay (ELISA) [1], radioimmunoassay (RIA) [2], fluorescence methods [3], and chemiluminescence [4]. Unfortunately, these standard methodologies have high cost, long analysis times (around 18 h) and require pretreatment of samples [5–6]. Other approaches to produce immunosensors have therefore been studied, including electroanalytical methods [7–8] in which antibodies or antigens are immobilized on a suitable matrix and the specific antigen–antibody recognition leads to an electrical or electrochemical signal.

The choice of the molecular architecture for the electrochemical immunosensors is crucial for obtaining high sensitivity and specificity. The matrix on which the bioactive layer is deposited may contain metallic nanoparticles to enhance the electrochemical response [8–9], including magnetic nanoparticles (MNPs) that can be exploited for their catalytic properties [10] and magnetic separation in pre-concentrating the analyte [11–16]. The most common magnetic nanoparticles used for this purpose are magnetite (Fe_3_O_4_) nanoparticles, which have a stronger magnetism than other iron oxide nanoparticles [17]. These MNPs can be synthesized through various techniques, such as ultrasound irradiation, sol–gel methods, thermal decomposition, and co-precipitation [18–21]. In addition, they can be modified with biomolecules and other compounds to improve the sensing performance. Electrochemical immunosensors containing magnetic nanoparticles have been used to detect several cancer biomarkers [22–24]. Zhuo and co-workers detected carcinoembryonic antigen (CEA) and α-fetoprotein (AFP) with a three-layer immunosensor with Fe_3_O_4_ magnetic core modified with a Prussian blue (PB) interlayer and a gold shell. The enzymes horseradish peroxidase and glucose oxidase were immobilized to improve sensitivity, with linear ranges between 0.01 and 80.0 ng·mL^−1^ for CEA and from 0.014 to 142 ng·mL^−1^ for AFP, and detection limits of 4 pg·mL^−1^ and 7 pg·mL^−1^, respectively [25]. PSA and interleukin 6 (IL-6) were measured with a microfluidic electrochemical immunoassay system, in which commercial magnetic particles were conjugated with secondary antibodies and horseradish peroxidase (HRP) [26]. These immunomagnetic nanoparticle-based microfluidic sensors with screen-printed carbon electrodes (INμ-SPCEs) showed limits of detection of 0.23 pg·mL^−1^ for PSA and 0.30 pg·mL^−1^ for IL-6, measured in the serum of prostate cancer patients [26]. Immunosensors to detect PSA include magnetic nanoparticles modified with gold [27], nitrodopamine functionalized iron oxide nanoparticles [3,28], ferrocene [28] and others [29–30].

A major challenge regarding the use of immunosensors in real samples lies in the difficulty to analyze considerable amounts of data in a single analysis, especially owing to the expected variability of blood serum, saliva, urine and tissue samples. This has sparked interest in computational tools [31]. For instance, information visualization techniques have been used to enhance the distinguishing ability of biosensors [32–34]. Discrimination of blood serum samples from patients with distinct probability to develop pancreatic cancer was made possible with a multidimensional projection technique applied to immunosensing data [34]. In this paper, we describe an INμ-SPCE to detect PSA using amperometry. To the best of our knowledge, the limit of detection is the lowest in the literature. The high sensitivity is probably connected to the molecular architecture of the sensing device, in which polyclonal antibodies were immobilized onto magnetic nanoparticles to selectively capture PSA. Furthermore, a proof-of-principle experiment regarding the detection of PSA in healthy and prostate cancer cell lysates is demonstrated, where the data were discriminated using multidimensional projections within the PEx-Sensors software [32].

## Results and Discussion

### Analytical performance

**Figure 1:**
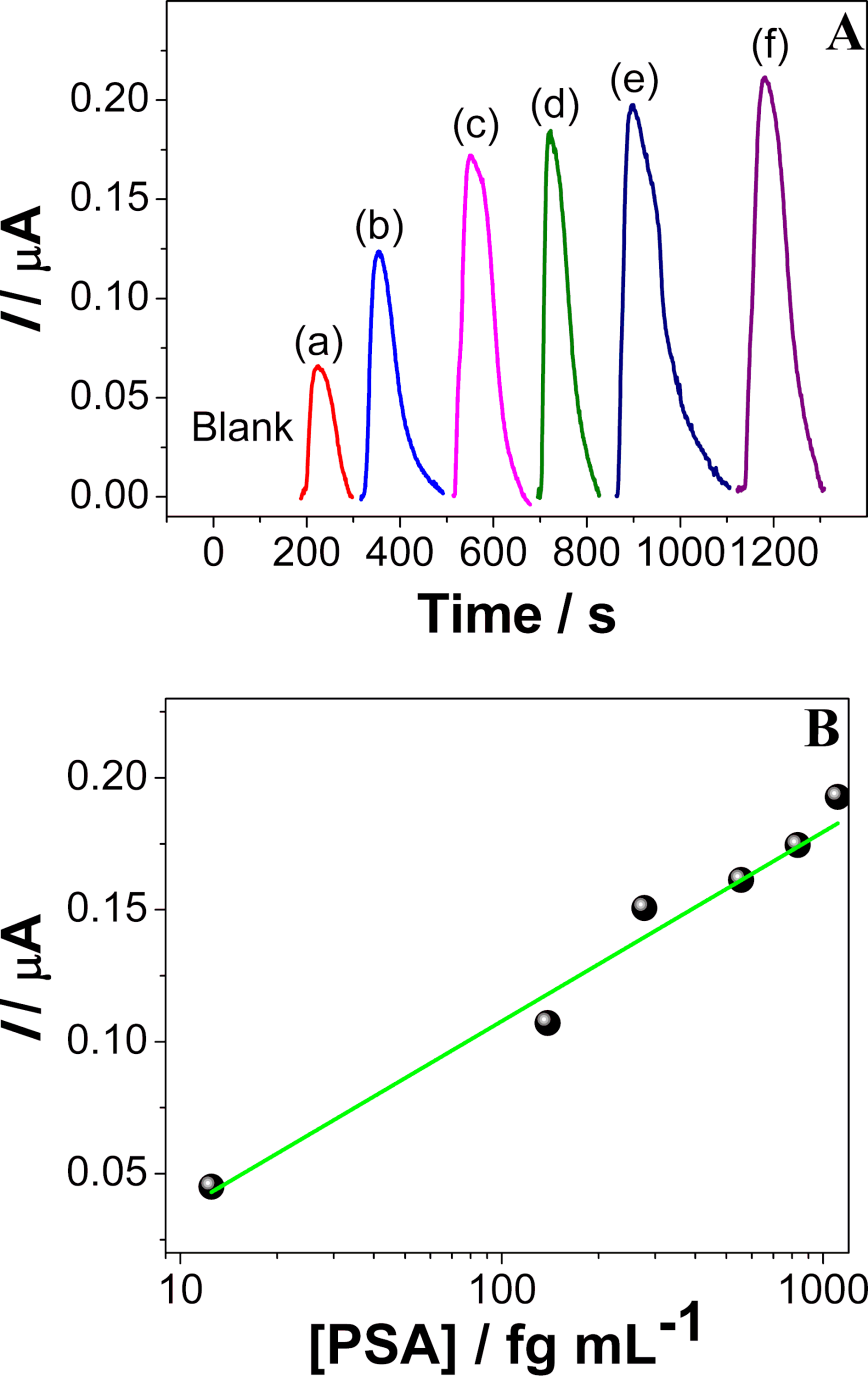
(A) Amperometric responses for a blank solution and PSA solutions at concentrations of: (a) 12.5, (b) 138, (c) 277, (d) 555, (e) 823 and (f) 1111 fg·mL^−1^ prepared in a calf serum medium. (B) Analytical curve for PSA standards, using MNPs, anti-Ab_1_ and anti-Ab_2_ at a concentration of 10 μg·mL^−1^, and concentrations of 10 µmol·L^−1^ and 1 μmol·L^−1^ for HQ and H_2_O_2_, respectively.

The analytical performance of the INμ-SPCEs was evaluated using PSA standard solutions in PBS at concentrations ranging from 12.5 to 1111 fg·mL^−1^. After capturing PSA with the bioconjugate (Ab_2_-MNP-HRP) from the standard solution, 100 µL were injected into the microfluidic channel using an injection valve, and then incubated for 30 min on an anti-PSA-AuNP-SPCE surface. A sandwich-type structure was assembled following the incubation. The amperometric analytical signal was obtained using constant-potential amperometry at a working electrode potential of −200 mV vs pseudo-reference Ag/AgCl. The mixed solution containing H_2_O_2_ (1 µmol·L^−1^) and hydroquinone (HQ, 10 µmol·L^−1^) was injected into the electrochemical cell, and the signal was monitored. The HRP-Fe(III) immobilized on the MNPs was oxidized by H_2_O_2_ to form an intermediate (Fe^4+^=O) and a porphyrin π-cation radical. The oxidized HRP was reduced by the mediator hydroquinone (HQ) forming benzoquinone (BQ), which was electrochemically reduced by accepting one electron from the electrode, with the enzyme returning to its native form. Figure 1A shows the cathodic peak current responses, with a linear dependence of the current on the logarithm of the PSA concentration in Figure 1B, according to the linear regression equation: *I*(nA) = 1.03·10^−6^ + 7.2·10^−8^ log(*x*). The detection limit calculated using the IUPAC method [35–36] in Equation 1 is 0.062 fg·mL^−1^ :

[1] $$\text{LD}=\frac{3\cdot \text{SD}}{\text{slope}},$$

where SD and slope are the standard deviation and slope of the calibration curve, respectively. To our knowledge, this limit of detection is the lowest found in the literature for immunosensors to detect PSA. The high sensitivity could be attributed to the use of MNPs (see characterization in Figure S1, Supporting Information File 1) decorated with Ab2 and HRP, which allowed for the capture, separation, and preconcentration of the analyte. It helped to acquire an amplified signal response and assisted the binding capacity of the antibody and antigen covalently immobilized on the electrode surface. Also, the monoclonal antibody provides high specificity to a single epitope, which is reflected in a low cross-reactivity. A comparison of various sandwich-type immunosensors and immunoassays for detection of PSA in the literature is presented in Table 1.

**Table 1:** Comparison of various sandwich-type immunosensors and immunoassays for the detection of PSA.

measurement method	linear range	detection limit	reference

amperometry	2–15 ng mL^−1^ and 15–120 ng mL^−1^	1.1 ng·mL^−1^	[37]
electrochemical impedance spectroscopy (EIS)	1–100 pg·mL^−1^	1 pg·mL^−1^	[38]
linear sweep voltammetry (LSV)	1–35 ng·mL^−1^	0.76 pg·mL^−1^	[39]
LSV	1–10 ng·mL^−1^	1 ng·mL^−1^	[40]
chemiluminescence	0.74 pg·mL^−1^ to 0.74 µg·L^−1^	0.7 pg·mL^−1^	[41]
amperometry	0–60 µg·L^−1^	0.08 µg·L^−1^	[5]
surface plasmon resonance	1–100 ng·mL^−1^	1 ng·mL^−1^	[42]
chronoamperometry	1 × 10^−5^ ng·mL^−1^ to 100 ng·mL^−1^	0.002 pg·mL^−1^	[43]
amperometry	50 fg·mL^−1^ to 40 ng·mL^−1^	16.6 fg·mL^−1^	[44]
EIS	1 pg·mL^−1^ to 100 ng·mL^−1^	1 pg·mL^−1^	[45]
EIS	0–10 ng·mL^−1^	590 pg·mL^−1^	[46]
EIS	0.05–5 ng·mL^−1^	13 pg·mL^−1^	[47]
EIS	0.01–10 ng·mL^−1^	2 pg·mL^−1^	[48]
chip enzyme immunoassay	3.2–50 ng·mL^−1^	3.2 ng·mL^−1^	[49]
electrochemical chemiluminescence (ECL)	0.0001–100 ng·mL^−1^	0.1 pg·mL^−1^	[50]
differential pulse voltammetry (DPV)	0.001–5 ng·mL^−1^	0.31 pg·mL^−1^	[51]
EIS	0.5 pg·mL^−1^ to 35 ng·mL^−1^	5 pg·mL^−1^	[52]
DPV	0.1 pg·mL^−1^ to 90 ng·mL^−1^	10 fg·mL^−1^	[52]
DPV	0.2–40 ng·mL^−1^	0.020 ng·mL^−1^	[53]
amperometry	12.5–1111 fg·mL^−1^	0.062 fg·mL^−1^	this work

Repeatability is a significant parameter for immunosensors. It was checked by using eight working electrodes in an array prepared under the same conditions, with the electrochemical response obtained at a given PSA concentration. The relative standard deviation in percent varied from 6% to 9%, with similar electrochemical responses for all immunosensors.

**Figure 2:**
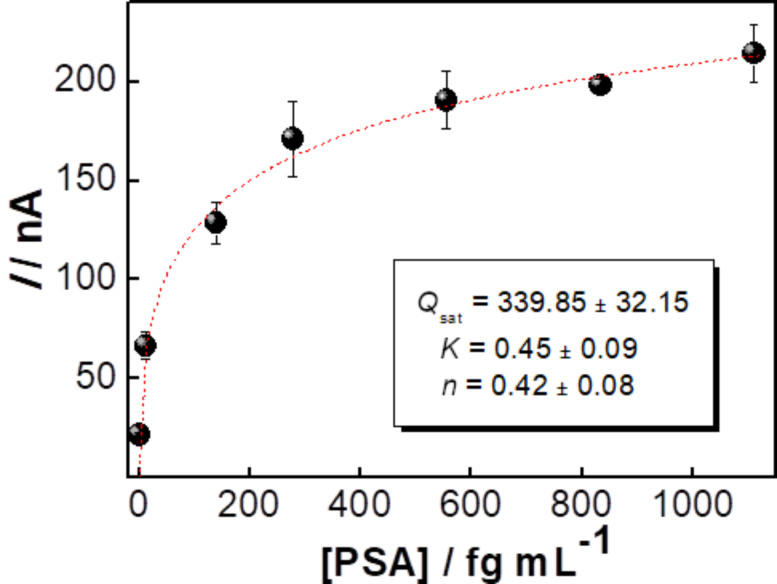
Peak current as a function of the PSA concentration fitted with a Langmuir–Freundlich equation (dashed line).

The detection mechanism for the immunosensors is likely an adsorption process, which is common for this type of sensor. We verified this hypothesis by modeling the amperometric responses for PSA antigen at concentrations from 12.5 to 1111 fg·mL^−1^ in Figure 2, where a Langmuir–Freundlich equation (Equation 2) was used to fit the data:

[2] $$q=\frac{Q_{m}{\left(K_{\text{a}}C_{\text{eq}}\right)}^{n}}{{\left(K_{\text{a}}C_{\text{eq}}\right)}^{n}+1},$$

where *Q_m_* is the adsorption capacity in nA, *K*_a_ is the adsorption affinity constant in milliliter per femtogram, *C*_eq_ is the concentration of the analyte in solution and *n* is a dimensionless index of heterogeneity, which varies between 0 and 1 for heterogeneous materials (*n* = 1 for homogeneous materials) [54]. Figure 2 shows the results with saturation of available sites with *Q_m_*= 339.85 ± 32.15 nA, which corresponds to ca. 64.4 fg·mL^−1^, *n* = 0.42 ± 0.08 and an affinity constant (*K*_a_ = 0.45 ± 0.09 mL·fg^−1^) characteristic of a polymer-based immunosensor [34,55]. This rather low value is expected for biosensors where the index *n* is characteristic of heterogeneous adsorption with polyclonal biomolecules that have many active sites with different degrees of affinity and selectivity.

### Application of the immunosensor in real samples

**Figure 3:**
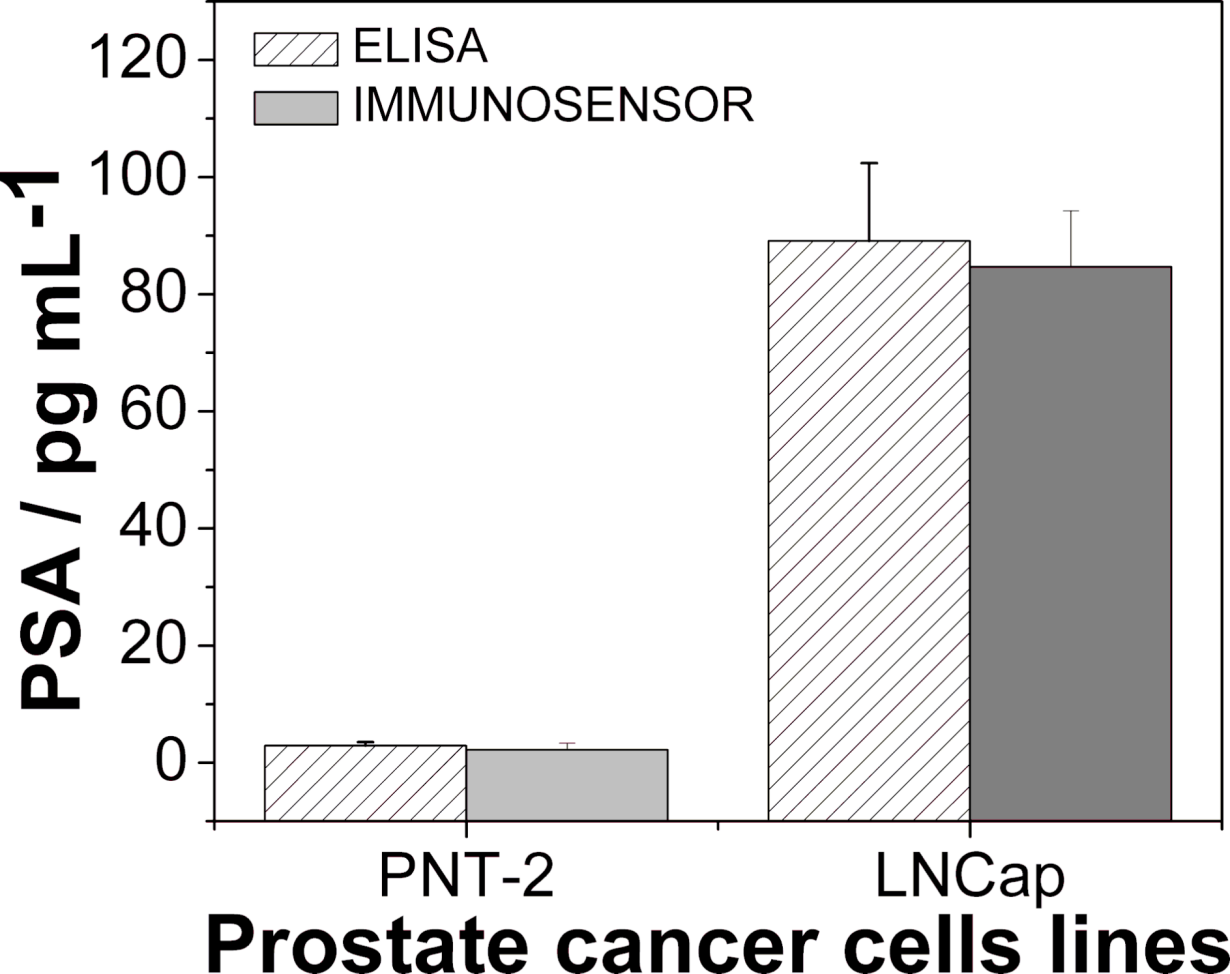
ELISA and INμ-SPCE results for PSA in control cell lysates (PNT-2) and prostate cancer cells (LNCap).

The suitability of INμ-SPCEs for detecting PSA in real samples was tested with malignant (LNCap) and non-malignant (PNT-2) cells. In contact with cell lysates containing several proteins, the bioconjugate binds specifically to PSA (PSA-Ab_2_-MNP-HRP), thus allowing for the capture, separation and preconcentration of PSA employing a magnet. Furthermore, detection is enhanced because of the presence of multiple immobilized HRP molecules. Figure 3 shows that a high amount of PSA is found in LNCap in comparison to PNT-2 using the immunosensor, in agreement with the standard ELISA method. The limit of quantification with the immunosensor is lower than the threshold established for the serum level found in patients with prostate cancer (above 3.6 ng·mL^−1^ stage A1) [56]. The samples were diluted in PBS for reaching the linear range, providing a response within the stipulated standards for the samples. Using the linear discrimination technique, the concentration is predicted for the real samples with 91.67% accuracy.

### Information visualization applied to the immunosensing data

**Figure 4:**
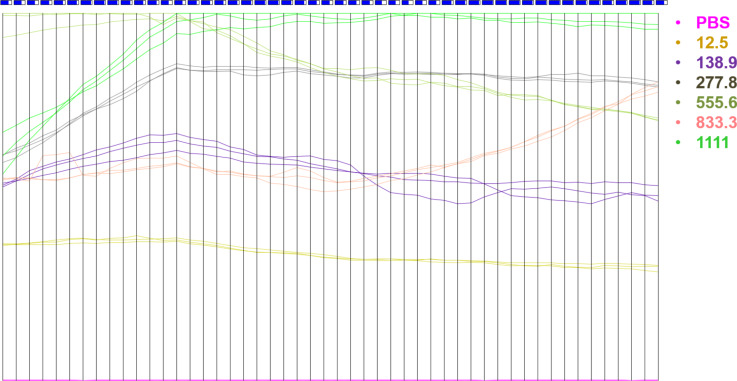
Parallel coordinates plot for PSA concentrations from 12.5 to 1111 fg·mL^−1^ after the feature-selection procedure. The *x*-axis represents time values, while the *y*-axis represents Euclidean distances related to the current.

The sensitivity of the INμ-SPCEs could be exploited in distinguishing a diversity of samples by using multidimensional projection techniques. The whole amperograms in Figure 1A were processed with four multidimensional projection techniques, namely, principal component analysis (PCA), least square projection (LSP), interactive document mapping (IDMAP) and Sammon’s mapping (SM), and the silhouette coefficients, *S*, were calculated as summarized in Figure S2 (Supporting Information File 1). The samples can be discriminated very well in all cases because *S* > 0.71 [46], and the highest value was obtained with the IDMAP technique. From the parallel coordinates (PC) plot in Figure S3 (Supporting Information File 1), we notice that the initial values for the current hamper discrimination, and therefore these dimensions (corresponding to times) are marked as red boxes (i.e., *S* < 0) in the upper part of the map. To improve discrimination, we adopted a feature-selection procedure [22] that consists in eliminating the dimensions that hamper discrimination. Figure 4 shows the parallel coordinates plot after feature selection, which leads to clear discrimination where the dimensions all contribute to detection, as represented by the blue boxes (i.e., *S* > 0).

**Figure 5:**
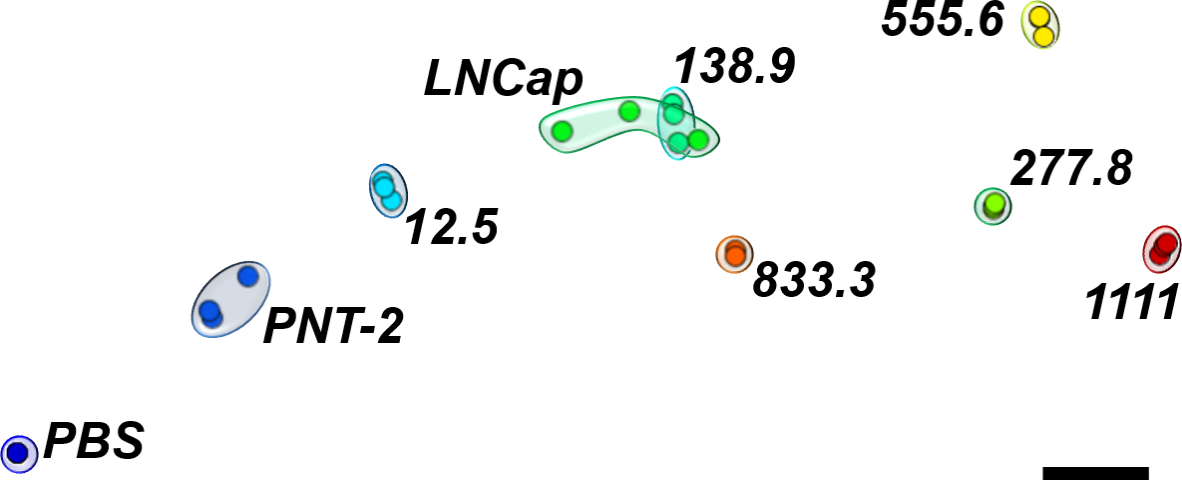
IDMAP plot obtained from the data in Figure 1A for buffers containing different PSA concentrations and from Figure 3 for prostate cancer cells. In both cases, feature selection was applied before plotting the data.

The maps obtained with the various multidimensional projection techniques after feature selection are shown in Figure S4 (Supporting Information File 1). The silhouette coefficients *S* increased by about 20% in comparison to the values without feature selection. The *S* value for IDMAP was calculated using the following equation:

[3] $$S_{\text{IDMAP}}=\frac{\delta \left(X_{i},X_{j}\right)-\delta_{\min}}{\delta_{\max}-\delta_{\min}}-\delta \left(Y_{i},Y_{j}\right),$$

where the minimum and maximum distances between the concentration values are given as δ_min_ and δ_max_, respectively, and δ(*X_i_*, *X_j_*) is the Euclidean distance between current responses for the PSA concentrations *X_i_* and *X_j_* [32]. IDMAP was found to give the highest *S* values and was used to project the data in Figure 5. One should note the large distance between the data points for PBS and those for the smallest concentration tested. This means that it is probably possible to detect PSA concentrations even lower than 12.5 fg·mL^−1^. The projection is consistent with the PSA concentrations obtained with ELISA for PNT-2 and LNCap cells with values of 5 and 84–92 fg·mL^−1^, respectively. This can be seen by the location of the sandwich-type immunosensing data for these cells in Figure 5.

## Conclusion

In this paper, we leverage sensing technologies to achieve ultrahigh sensitivity in detecting the prostate cancer biomarker PSA by using MNPs to capture PSA in a pre-concentration procedure for a sandwich-type immunomagnetic sensor. Electrochemical immunoassays with disposable microfluidic devices led to excellent linearity, reproducibility, and fast detection at low-cost, while showing excellent agreement with the standard method ELISA. Importantly, the approach for the immunoassays can be adapted for multiplex detection of biomarkers, in addition to PSA, by combining with other proteins. We also demonstrated that information visualization techniques, so far most commonly used for impedance spectroscopy sensing data, can be applied to amperometric results with a microfluidic sandwich-type immunosensor. The data processing allows for a more didactic interpretation of the results and improves separation between samples of patients with high levels of PSA from those who have lower concentrations.

## Experimental

### Materials

The reagents used were either of analytical or HPLC grade. Reduced ʟ-glutathione (GSH, 99%), bovine serum albumin (BSA), HAuCl_4_·3H_2_O (99.9%), sodium borohydride (99%), horseradish peroxidase (HRP, *M*_W_ = 44000; 250–330 u·mg^−1^), poly(diallydimethylammonium chloride) (PDDA, 20 wt % in H_2_0), 1-(3-(dimethylamino)propyl)-3-ethylcarbodiimide hydrochloride (EDC), *N*-hydroxysulfosuccinimide (Sulfo-NHS), hydrogen peroxide (H_2_O_2_, 30%), Tween-20, and 2-(*N*-morpholino)ethanesulfonic acid hydrate (MES) were purchased from Sigma-Aldrich. Monoclonal (mouse) primary anti-human prostate specific antigen (PSA) antibody, natural human prostate-specific antigen (PSA), and standard and secondary anti-PSA antibodies were obtained from Abcam, Cambridge, UK. Graphite-based ink was obtained from Henkel Electrodag, USA (reference code 423SS), silver chloride ink was purchased from Gwent electronic materials Ltd., UK (Product code C2130905D3), the cell lines PNT-2 and LNCap were acquired from the Banco de Células do Rio de Janeiro (BCRJ) (Rio de Janeiro, Brazil). For the synthesis of magnetic nanoparticles, sodium hydroxide (NaOH) with 97% purity and ferrous sulfate heptahydrate (FeSO_4_·7H_2_O) with ≥99.6% purity were purchased from Vetec Química Fina Ltda (Rio de Janeiro, Brazil), and ferric chloride hexahydrate (FeCl_3_·6H_2_O) with ≥98% purity was purchased from Sigma-Aldrich. Hydrochloric acid (HCl, 36.5–38.0% w/w) and NaCl with 99% purity were acquired from Labsynth (São Paulo, Brazil). Sodium citrate (Na_3_C_6_H_5_O_7_) with 99.8% purity was purchased from J.T Baker Chemical Company. All aqueous solutions were prepared with ultrapure water (18 MΩ·cm at 25 °C) obtained from a Milli-Q Direct-0.3 (Millipore) purification system.

### Fabrication of sandwich-type electrochemical immunosensors

**Figure 6:**
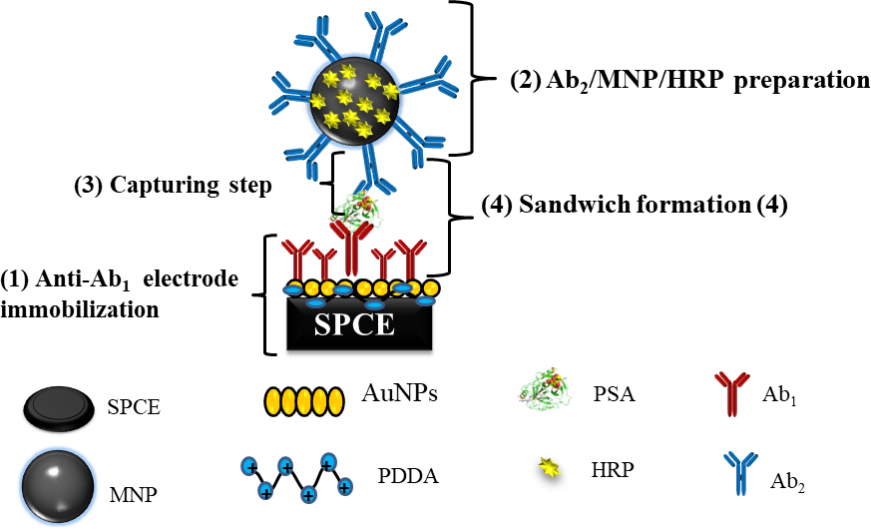
Schematic illustration of the fabrication of sandwich-type electrochemical immunosensors (INμ-SPCEs).

The multi-channel screen-printed array of electrodes was fabricated according to the procedures established by Faria and collaborators [57]. The experimental details are given in Supporting Information File 1. The fabrication of this sandwich-type immunosensor comprises four steps, as depicted in Figure 6: (1) deposition of monoclonal antibody on the carbon electrode, (2) bioconjugate modification using HRP and polyclonal antibody, (3) capture of biomarker by the bioconjugate, (4) sandwich formation by injection of the biomarker captured by Ab_2_-MNP-HRP in the microfluidic system.

**Figure 7:**
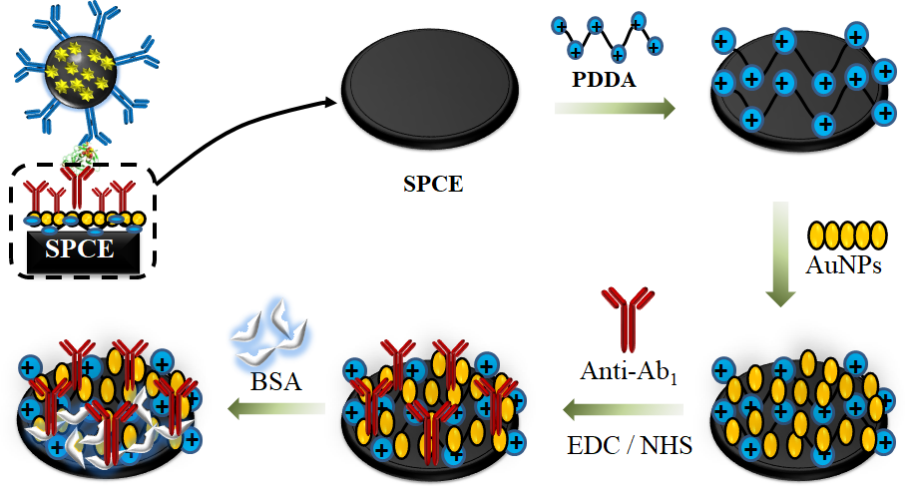
Electrode modification with 10 μg·mL^−1^ monoclonal antibody (Ab_1_) using 5 μL of 2 mg·mL^−1^ PDDA and 5 μL of AuNP-GSH. To activate the carboxyl groups from AuNP-GSH and ensure a stable covalent binding of antibodies, EDC/NHS was used (0.4 mol·L^−1^ EDC and 0.1 mol·L^−1^ NHS). The electrodes were washed with 1.0 mL of PBS buffer pH 7.4 and incubated with 5 μL of bovine serum albumin (BSA) (2% w/w) diluted in phosphate-buffered saline (PBS), to avoid non-specific binding.

The first step included the deposition of a bilayer of poly(diallyldimethylammonium) (PDDA) and gold nanoparticles (AuNPs) decorated with glutathione, as illustrated in Figure 7. An aliquot of 5 μL of 2.0 mg·mL^−1^ of PDDA was added to each working electrode and kept for 20 min. The electrode surface was rinsed with Millipore water to remove excess of reagents. Then, 5.0 μL of gold nanoparticles modified with glutathione (AuNP-GSH, 46.68 µg·mL^−1^) were dripped on the electrode and left for a period of 20 min. The carboxyl-terminated AuNP-GSH provided a chemical group suitable for covalent binding. Next, a volume of 5 μL EDC/NHS (0.4 mol·L^−1^ EDC and 0.1 mol·L^−1^ NHS) in water was added on the surface of INμ-SPCEs and kept for 10 min to activate the carboxyl groups from AuNP-GSH, therefore ensuring a stable covalent binding to the antibodies. The primary monoclonal antibodies (Ab1) were adsorbed on the electrode by adding 5 μL of a 10 μg·mL^−1^ solution in PBS 7.0, with adsorption occurring overnight. The electrodes were then washed with 1.0 mL PBS and incubated with 5 μL of bovine serum albumin (BSA) (2% w/w) diluted in PBS to avoid non-specific binding (NBS). Each step of the modification was monitored with polarization-modulated infrared reflection absorption spectroscopy (PM-IRRAS, see Figure S1F in Supporting Information File 1). The microfluidic cell was set up, and the electrodes were insulated using a polystyrene card with double-sided adhesive. The double-sided tape was used to delimit the electroactive area, which was fixed on the reference electrode under the arrangement of working electrodes and the auxiliary electrode. Also, the double-sided tape allowed for sealing of the microfluidic system.

The second step of the preparation of the sandwich immunosensors consisted in forming the bioconjugate complex of Ab_2_ and HRP (Ab_2_-MNP-HRP), as described by Uliana and co-workers [58]. Briefly, 2.0 mL (stock solution: 10 mg particles·mL^−1^) of MNPs were placed in microtubes, which were then washed with 500 μL of 0.05 mol·L^−1^ MES buffer at pH 5.2 and separated magnetically. Later, the supernatant was discarded and washed thrice to give a final particle concentration of 40 mg·mL^−1^. A 3 mg·mL^−1^ solution of EDC/NHS in 0.05 mol·L^−1^ MES buffer at pH 5.2 was added to the washed MNPs. It was shaken on a vortex-type stirrer for 5 min and on a rotary shaker for 30 min. Immediately after shaking, the particles were magnetically separated and washed with MES buffer again. The supernatant was then removed, and the washing procedure was repeated twice again. Subsequently, 250 μL of Ab_2_ were added to the solution with a final concentration of 10 μg·mL^−1^. The solution was vortexed and shaken on a custom-made rotary shaker for 24 h at room temperature. At this point, the solution was magnetically separated and washed with 600 μL PBS/0.05% Tween 20 buffer pH 7.4.

**Figure 8:**
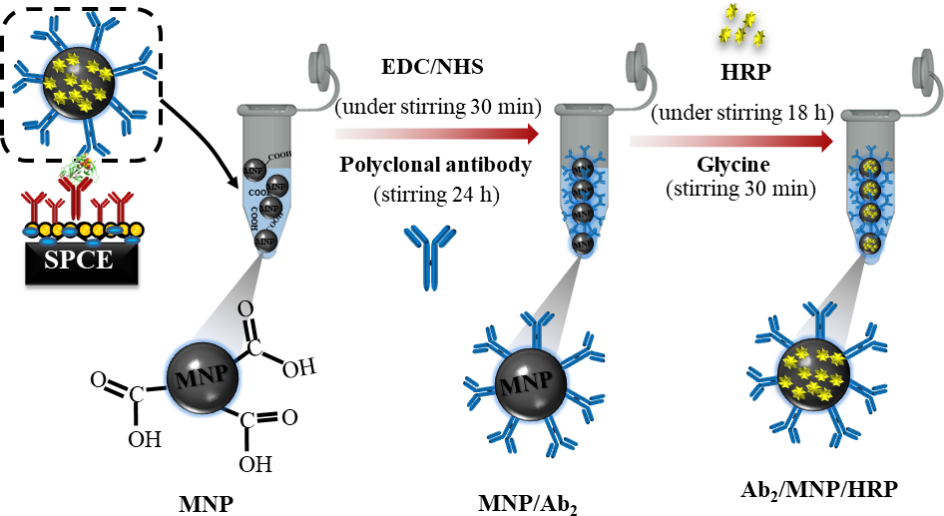
Preparation of the bioconjugate complex of Ab_2_ and HRP (Ab_2_-MNP-HRP).

HRP was conjugated to the MNPs by adding 500 μL of 1.2 mg·mL^−1^ of the enzyme to the Ab_2_-MNP complex. The mixture was left overnight on a rotary shaker. After 18 h of stirring, the bioconjugate complex was magnetically separated and then washed with 600 μL of PBS/0.05% Tween-20 and 0.1% BSA (four-fold). Later, 1.0 mL of glycine 1.0 mol·L^−1^ pH 8 was added to the solution. The solution was vortexed and stirred for 30 min at room temperature, followed by washing with 0.05% PBS/Tween-20 buffer, pH 7.4 and 2% BSA; and magnetic separation. Finally, the bioconjugate complex was resuspended with 250 μL of 0.05% PBS-Tween/20 buffer of pH 7.4 and 2% BSA giving a final particle concentration of 5 mg·mL^−1^. These steps are summarized in Figure 8.

**Figure 9:**
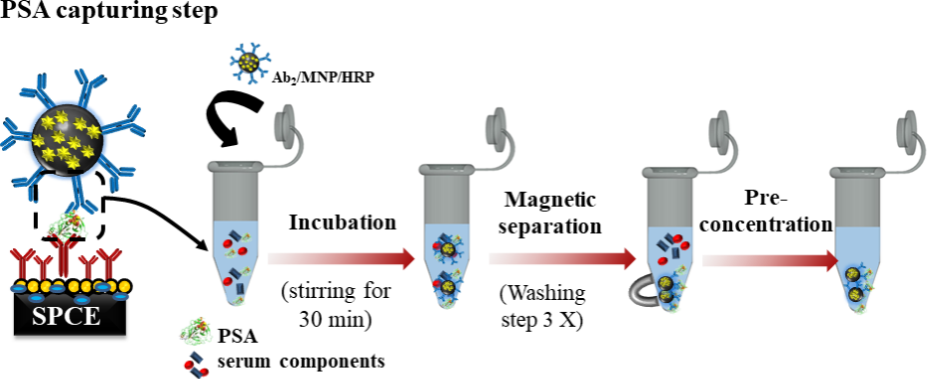
Illustration of the PSA capturing step.

In the third step, shown in Figure 9, 20 μL of Ab_2_-MNP-HRP were added to 320 μL PBS buffer at pH 7.4. For the standard calibration, 20 μL of the antigen-enriched calf serum were added to the composite bioconjugate complex mixture. The mixture was then incubated at 37 °C for 30 min, and dilutions were required to decrease the protein concentration. The devices were also evaluated with real samples, including culture medium of cancerous and control cells (lineage of LNCap and PNT-2 cells, respectively). The cell lines PNT-2 and LNCap were acquired from the Banco de Células do Rio de Janeiro (BCRJ, Rio de Janeiro, Brazil). The samples were diluted to a 1:30000 ratio. The resulting conjugate, Ab_2_-MNP-HRP-protein, was magnetically separated and washed with 400 μL of 0.5% BSA and 20 mol·L^−1^ of PBS buffer pH 7.4. It was then resuspended to 125 μL.

In the fourth and last step, the bioconjugate complex was used to form the sandwich-type immunosensor, with 125 μL of the final solution used in the immunoassay to fill a 100 μL sample loop. This was performed in a microfluidic system with a flow rate of 100 µL·min^−1^. The complex was injected with a syringe and the flow was stopped for 30 min. In the amperometric detection step, the biomarker quantification was performed by an indirect method through the electrochemical response of the marker in the MNPs. It was monitored by injecting a solution containing 1.0 μmol·L^−1^ of H_2_O_2_ and 10 μmol·L^−1^ of hydroquinone (HQ) in the microfluidic system. The amperometric measurements were performed using a DropSens μStat 8000 multi-potentiostat/galvanostat. The multichannel screen-printed array contained eight working carbon electrodes combined with one pseudo-reference electrode (Ag/AgCl) and one auxiliary electrode (carbon). The microfluidic system was set up with an injection pump (NE-1000 programmable Single Syringe Pump, New Era Pump System, Farmingdale, USA) and an injection valve with a sample loop of 100 µL.

### Data treatment with information visualization methods

The distinction of real samples is certainly challenging when various samples are analyzed, and false positives may occur. This has prompted researchers to use statistical and computational methods to treat the sensing data [31], in some cases with conjunction with machine learning approaches [31,59]. Here we employed multidimensional projection techniques based on linear and nonlinear multidimensional scaling (MDS) approaches such as principal component analysis (PCA) [60], Least squares projection (LSP) [61], Sammon’s mapping (SM) [62] and interactive document mapping (IDMAP) [63] implemented in the software as projection explorer sensors (PEx-Sensors) [32,64]. The amperogram data (current as a function of the time) were dimensionally reduced by PCA and FastMap [65] and then projected with PCA, LSP, IDMAP, and SM techniques. The dissimilarities between samples were converted to Euclidean distances with the whole rising current curves being transformed into single data points as observed in the projection plots. Three independent sets of measurements were utilized on this analysis. A full description of these techniques and PEx-sensors can be found in [32].

This type of analysis provides a map for pattern recognition among samples. It has been applied to biosensing data, mainly with IDMAP, which includes an algorithm to minimize the global error through a pairwise error function [63]. Herein, we combined the projections with parallel coordinates maps to perform feature selection with the exclusion of dimensions found to be deleterious for discrimination of similar data points, analogously to [66]. The performance upon applying the different projection techniques was evaluated by calculating the silhouette coefficient, *S*, defined as the average of the distances between each data instance and all other points belonging to the same group, and the minimum distance between each data instance and other instances belonging to other groups [67]. *S* values vary between −1 and 1. According to Rousseeuw, values above 0.71 indicate that a strong discrimination was obtained [68].

## Supporting Information

Supporting Information features detailed information on the synthesis of magnetic iron oxide nanoparticles, electrode fabrication, and sample preparation. Also, the characterization of MNPs and electrode surfaces by using Fourier-transform infrared spectroscopy (FTIR), field-emission scanning electron microscopy (FE-SEM), energy-dispersive X-ray spectroscopy (EDX), and polarization-modulated infrared reflection absorption spectroscopy (PM-IRRAS, Figure S1) is described. The Silhouette coefficients calculated for IDMAP, Sammon’s mapping (SM), principal component analysis (PCA), and least square projection (LSP) multidimensional projection techniques to analyze the PSA concentration of immunosensor before (All) and after (FS) feature selection (Figure S2) are provided as well. The parallel coordinates plot for PSA concentrations from 12.5 to 1111 fg·mL^−1^ are given in Figure S3, while plots of three multidimensional projection techniques, i.e., PCA, LSP, and SM are shown in Figure S4.

File 1 Additional procedures and figures.
