# Supplementary material for: Use of data processing for rapid detection of the prostate-specific antigen biomarker using immunomagnetic sandwich-type sensors
Source: Beilstein J Nanotechnol. 2019 Nov 6;10:2171–81. doi: 10.3762/bjnano.10.210 (PMC6880837; doi:10.3762/bjnano.10.210)
Supplement: File 1 — Additional procedures and figures. [file Beilstein_J_Nanotechnol-10-2171-s001.pdf]

BEILSTEIN JOURNAL OF NANOTECHNOLOGY

Supporting Information

for

Use of data processing for rapid detection of the prostate-specific antigen biomarker using immunomagnetic sandwich-type sensors

Camila A. Proença, Tayane A. Freitas, Thaísa A. Baldo, Elsa M. Materón, Flávio M. Shimizu, Gabriella R. Ferreira, Frederico L. F. Soares, Ronaldo C. Faria and Osvaldo N. Oliveira Jr.

Beilstein J. Nanotechnol. **2019,** *10,* 2171–2181. doi:10.3762/bjnano.10.210

Additional procedures and figures

License and Terms: This is a supporting information file under the terms of the Creative Commons Attribution License (http://creativecommons.org/licenses/by/4.0). Please note that the reuse, redistribution and reproduction in particular requires that the authors and source are credited.
The license is subject to the *Beilstein Journal of Nanotechnology* terms and conditions: (https://www.beilstein-journals.org/bjnano)

Synthesis of magnetic iron oxide nanoparticles

The Fe_3O_4 magnetic nanoparticles (MNPs) were synthesized according to the co-precipitation method reported by Ferreira and co-workers [1], with modifications. The MNPs were synthesized using 1.55 g of $FeSO_4 \cdot 7H_2O$ and 3.05 g of $FeCl_3 \cdot 6H_2O$, dissolved in 2.5 mL of HCl, followed by dilution in 62.5 mL of distilled water and heating to 60 °C. A hot plate IKA C-MAG model HS 7 (IKA Works, Inc.), connected to an integrated temperature control and a Pt1000 temperature probe, was used to prepare the MNPs. A molar ratio of 1:2 between Fe^{2+} and Fe^{3+} was used during reaction. A solution with 18.75 g NaOH dissolved in 312.5 mL of H_2O in a beaker, heated to 60 °C, was mixed with the previous solution and kept under vigorous stirring. The reaction mixture was maintained at 60 °C for 30 min. The magnetic nanoparticle solution was washed repeatedly with distilled water until it reached pH 7 and was then separated by magnetic decantation. Then, 15 mL of this solution were removed and stored in acetone. The solution was left under N_2 atmosphere for 30 min and stored in a refrigerator for further characterization.

The surface modification of Fe_3O_4 magnetic particles was carried out by using sodium citrate. Initially, the MNP solution was heated to 85 °C under stirring for 30 min. Then, sodium citrate was added in excess to the solution under stirring and at constant temperature for another 30 min. The modified particles were washed several times with distilled water until pH 7 was reached. The resulting functionalized MNPs were decanted off using a neodymium magnet and suspended in acetone. Subsequently, the solution was maintained in N_2 atmosphere for 30 min and stored in a refrigerator for later use.

Fabrication of electrodes

The all-plastic carbon electrodes consist of eight working electrodes (WE), a pseudo-reference electrode (RE), and a counter electrode (CE), designed with Silhouette Studio v. 2.7.4 software. The electrodes were cut out from an adhesive vinyl sheet using an electronic craft cutter (Silhouette Cameo, Silhouette America, Inc.), and unwanted vinyl was removed with tweezers. The mask was used as a template that was transferred to a polyester sheet (transparency film for printers) with dimensions of 21.0 cm by 29.7 mm. Then, graphite-based carbon ink (Electrodag 423SS) was transferred carefully and heated at 90 °C for 30 min. The pseudo-reference electrode (Ag/AgCl) (C2051014P10, Gwent Electronic Materials Ltd., UK) was then painted and cured for another 30 min at 60 °C. Finally, the vinyl mask was removed from the polyester sheet.

Sample preparation

The PSA standard solution was prepared by diluting the PSA antigen standard product, which was obtained from Abcam to different concentrations with calf serum. The healthy and prostate cancer cell linage were obtained from the Cell Bank from Rio de Janeiro/Brazil.

Analysis with ELISA

The ELISA test was performed following the Abcam protocol. An ELISA plate containing a solution of 50 µL samples, PSA standards and an antibody cocktail were added to each well. The ELISA plate was shaken (400 rpm) and incubated for 1 h at room temperature. The wells were washed thrice with PBS/0.005% T20 and 50 µL of TMB substrate were added to each well and incubated for 10 min in the dark. Then, 45 µL of

stop solution was added to each well. The samples were analyzed on the Labtech LT-4000MS Microplate Reader, and the absorbance was determined at the wavelength of 450 nm.

Characterization of MNPs and electrode surfaces

Transmission electron microscopy (TEM) images were obtained using a JEOL model JEM 200CX operating at an accelerating voltage of 200 kV. The TEM images of MNPs revealed spherical particles with less than 10 nm diameter, according to the TEM image in Figure S1A. Field-emission scanning electron microscopy (FE-SEM) images were obtained using a Hitachi model S-4800. Energy-dispersive X-ray spectroscopy (EDX) was performed using Cu Kα radiation (λ = 1.5418 Å, 40 kV and 30 mA) in the range of $2° \leq 2\theta \leq 80°$ with steps of 0.05° at a rate of 0.25° min^{-1} (Figure S1B,C). Figure S1B shows the SEM images of agglomerates (insert in Figure S1B). The average diameters of the agglomerate (inserts of Figure 1B) are similar to the values reported elsewhere [2-4]. Figure S1C shows the diffractogram of Fe_3O_4 nanoparticles, with typical peaks of magnetite at 30°, 35°, 45.3°, 54.6°, 59.3° and 74.6°, corresponding to the (220), (311), (400), (422), (511) and (533) reflections, respectively. Magnetite has a crystalline structure of the spinel type [5-7]. Figure S1D shows the typical EDX spectra of the Fe_3O_4 nanoparticles. The samples exhibit very strong characteristic Kα and Kβ peaks of iron in the range from 6.40 to 8.06 keV. These results confirm that the particles consist mostly of iron oxide.

The functionalization using citrate was confirmed with Fourier-transform infrared spectroscopy (FTIR) measurements using a FTIR-4100 spectrometer (Jasco, Essex, UK). Characteristic citrate peaks of C=O stretching at 1626 cm^{-1} and of OH from COOH between 3012 and 3600 cm^{-1} (Figure S1E) were observed [8]. Polarization-modulated

infrared reflection absorption spectroscopy (PM-IRRAS) [8] spectra for the step-by-step monitoring of the electrode surface are shown in Figure S1F. The baseline correction was made by taking the spectrum of a blank carbon surface as reference. The measurements were performed with a KSV PMI 550 instrument (KSV Instruments Ltd., Helsinki, Finland). The PM-IRRAS signal consisted of the average of 120 scans over a period of 20 min controlled with IRRAS 1.0.5 software, and was obtained after deposition of each layer of the sensing units. PDDA contains nitrogen atoms bonded to hydrogen, which are responsible for the strong bands assigned to N–H deformation and the asymmetric stretching mode of $(CH_3)_2N^+$ at 1277 and 900 cm^{-1}, respectively. A C–N bond gave rise to the broad band at 1175 cm^{-1}. The carboxylic group in AuNP-GSH yielded a band at 1045 cm^{-1} assigned to C–OH. The C=O band at 1634 cm^{-1} is due to the stretching vibration of the ester carbonyl group, absent in the PDDA spectrum. Immobilization of anti-PSA introduced the bands at 1634 cm^{-1} and 1541 cm^{-1} assigned to C=O stretching and C–N bending modes, respectively. Also, the bands at 1688, 1634, and 1455 cm^{-1} can be assigned to the primary amide.

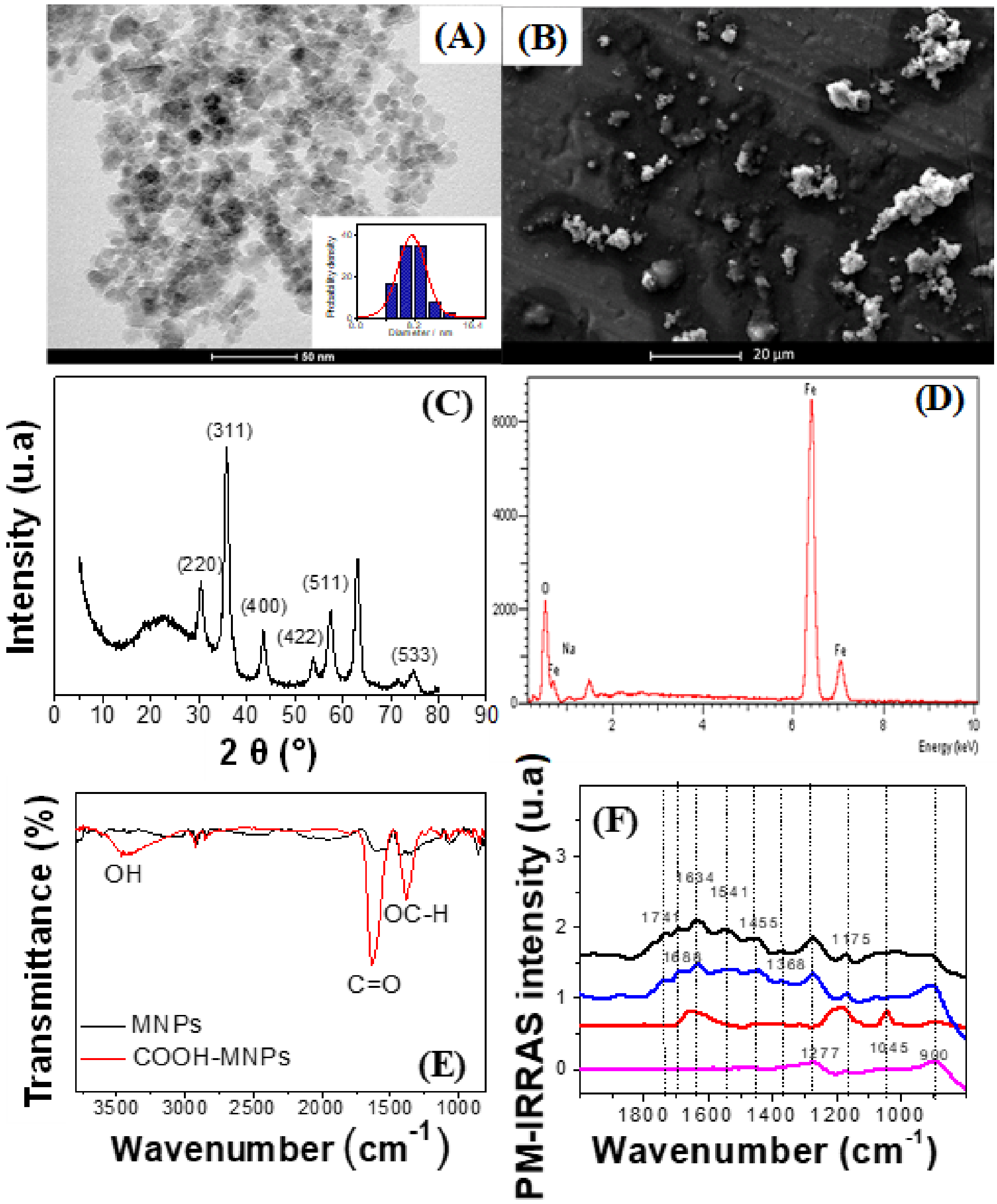

Figure S1: (A) TEM image of the synthesized MNPs and diameter distribution of MNPs (insert). (B) SEM image and diameter distribution of MNP agglomerates. (C) X-ray diffractogram and (D) energy-dispersive X-ray spectra of unmodified MNPs showing iron peaks. (E) Fourier-transform infrared (FTIR) spectra of unmodified MNPs. (F) PM-IRRAS spectra of immunosensor layers: SPCE/PDDA (pink line), SPCE/PDDA/Au/EDC-NHS (red line), SPCE/PDDA/Au/EDC-NHS (blue line) and SPCE/PDDA/Au/Ab2 (black line) in the region of 800–2000 cm^{-1}.

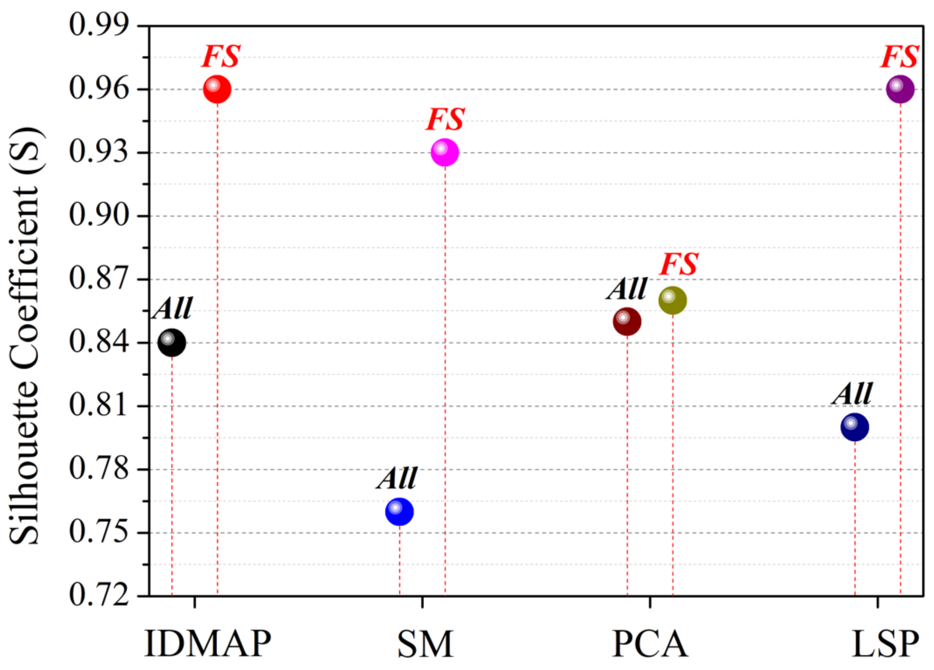

Figure S2: Silhouette coefficients calculated for IDMAP, Sammon's mapping (SM), principal component analysis (PCA), and least square projection (LSP) multidimensional projection techniques to analyze the PSA concentration before (All) and after (FS) feature selection.

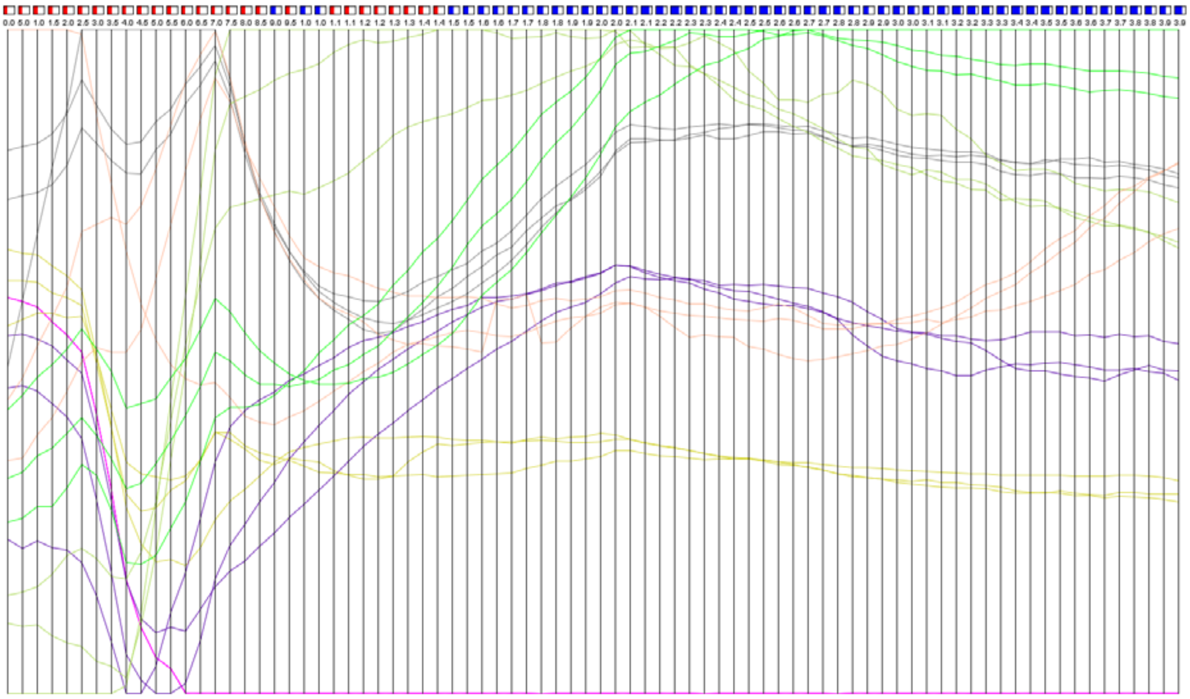

Figure S3: Parallel coordinates plot for PSA concentrations from 12.5 to 1111 fg·mL^{-1}.

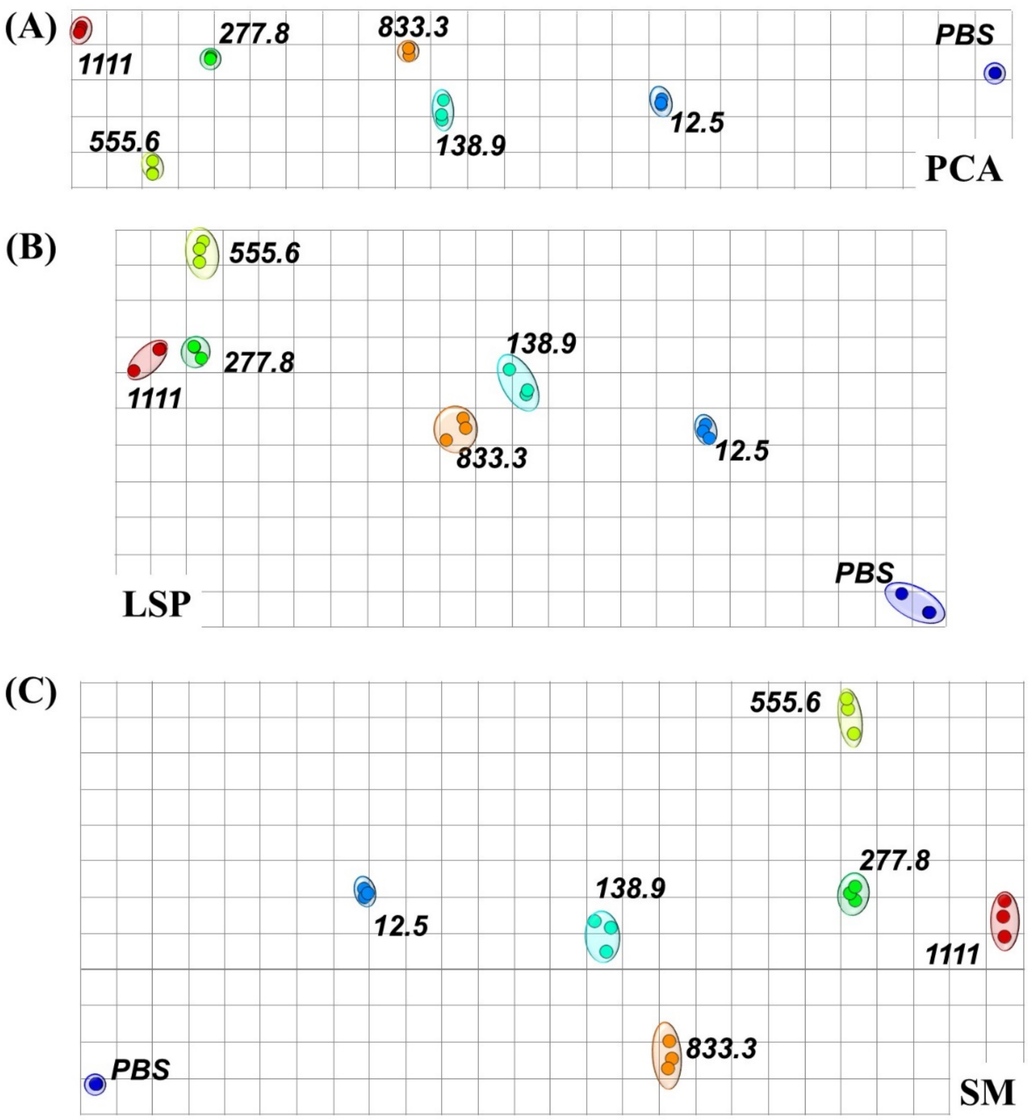

Figure S4: Plots obtained from the data in Figure 1A for buffers containing different PSA concentrations, using three multidimensional projection techniques: (A) PCA, (B) LSP, and (C) Sammon's mapping (SM).

References

1. Ferreira, G. R.; Segura, T.; de Souza, F. G.; Umpierre, A. P.; Machado, F. *Eur. Polym. J.* **2012,** *48*, 2050–2069. doi:10.1016/j.eurpolymj.2012.09.003

2. Valenzuela, R.; Fuentes, M. C.; Parra, C.; Baeza, J.; Duran, N.; Sharma, S. K.; Knobel, M.; Freer, J. *J. Alloys Compd.* **2009,** *488,* 227–231. doi:10.1016/j.jallcom.2009.08.087
3. Hariani, P. L.; Faizal, M.; Ridwan, R.; Marsi, M.; Setiabudidaya, D. *Int. J. Environ. Sci. Dev.* **2013,** *4,* 336–340. doi:10.7763/IJESD.2013.V4.366
4. Aqil, A.; Vasseur, S.; Duguet, E.; Passirani, C.; Benoît, J. P.; Roch, A.; Müller, R.; Jérôme, R.; Jérôme, C. *Eur. Polym. J.* **2008,** *44,* 3191–3199. doi:10.1016/j.eurpolymj.2008.07.011
5. Wang, X.; Tang, S.; Liu, J.; He, Z.; An, L.; Zhang, C.; Hao, J.; Feng, W. *J. Nanopart. Res.* **2009,** *11,* 923–929. doi:10.1007/s11051-008-9486-9
6. De Souza, F. G.; Marins, J. A.; Pinto, J. C.; De Oliveira, G. E.; Rodrigues, C. M.; Lima, L. M. T. R. *J. Mater. Sci.* **2010,** *45,* 5012–5021. doi:10.1007/s10853-010-4321-y
7. Neves, J. S.; De Souza, F. G.; Suarez, P. A. Z.; Umpierre, A. P.; MacHado, F. *Macromol. Mater. Eng.* **2011,** *296,* 1107–1118. doi:10.1002/mame.201100050
8. Bieri, M.; Bürgi, T. *J. Phys. Chem. B* **2005,** *109,* 22476–22485. doi:10.1021/jp052409m
